# Disinhibition in dementia related to reduced morphometric similarity of cognitive control network

**DOI:** 10.1093/braincomms/fcae124

**Published:** 2024-04-16

**Authors:** Lisanne M Jenkins, Ashley Heywood, Sonya Gupta, Maryam Kouchakidivkolaei, Jaiashre Sridhar, Emily Rogalski, Sandra Weintraub, Karteek Popuri, Howard Rosen, Lei Wang, Frontotemporal Lobar Degeneration Neuroimaging Initiative, Howard Rosen, Bradford C Dickerson, Kimoko Domoto-Reilly, David Knopman, Bradley F Boeve, Adam L Boxer, John Kornak, Bruce L Miller, William W Seeley, Maria-Luisa Gorno-Tempini, Scott McGinnis, Maria Luisa Mandelli

**Affiliations:** Psychiatry and Behavioral Sciences, Northwestern University, Chicago, IL 60611, USA; Psychiatry and Behavioral Sciences, Northwestern University, Chicago, IL 60611, USA; Psychiatry and Behavioral Sciences, Northwestern University, Chicago, IL 60611, USA; School of Engineering Science, Simon Fraser University, Burnaby, BC V5A 1S6, Canada; Mesulam Center for Cognitive Neurology and Alzheimer’s Disease, Northwestern University, Chicago, IL 60611, USA; Psychiatry and Behavioral Sciences, Northwestern University, Chicago, IL 60611, USA; Mesulam Center for Cognitive Neurology and Alzheimer’s Disease, Northwestern University, Chicago, IL 60611, USA; Psychiatry and Behavioral Sciences, Northwestern University, Chicago, IL 60611, USA; Mesulam Center for Cognitive Neurology and Alzheimer’s Disease, Northwestern University, Chicago, IL 60611, USA; Computer Science, Memorial University of Newfoundland, St. Johns, NL A1C 5S7, Canada; Neurology, University of California, San Francisco, CA 94143, USA; Psychiatry and Behavioral Health, Ohio State University, Columbus, OH 43210, USA

**Keywords:** behavioural variant frontotemporal dementia, Alzheimer’s dementia, morphometric similarity networks, disinhibition, MRI

## Abstract

Disinhibition is one of the most distressing and difficult to treat neuropsychiatric symptoms of dementia. It involves socially inappropriate behaviours, such as hypersexual comments, inappropriate approaching of strangers and excessive jocularity. Disinhibition occurs in multiple dementia syndromes, including behavioural variant frontotemporal dementia, and dementia of the Alzheimer’s type. Morphometric similarity networks are a relatively new method for examining brain structure and can be used to calculate measures of network integrity on large scale brain networks and subnetworks such as the salience network and cognitive control network.

In a cross-sectional study, we calculated morphometric similarity networks to determine whether disinhibition in behavioural variant frontotemporal dementia (n = 75) and dementia of the Alzheimer’s type (n = 111) was associated with reduced integrity of these networks independent of diagnosis.

We found that presence of disinhibition, measured by the Neuropsychiatric Inventory Questionnaire, was associated with reduced global efficiency of the cognitive control network in both dementia of the Alzheimer’s type and behavioural variant frontotemporal dementia.

Future research should replicate this transdiagnostic finding in other dementia diagnoses and imaging modalities, and investigate the potential for intervention at the level of the cognitive control network to target disinhibition.

## Teaser

Disinhibition occurs in multiple dementia syndromes, including behavioural variant frontotemporal dementia and dementia of the Alzheimer’s type. Jenkins *et al*. constructed Morphometric Similarity Networks and found that presence of disinhibition, measured by the Neuropsychiatric Inventory Questionnaire, was associated with reduced global efficiency of the cognitive control network, regardless of diagnosis.

## Graphical Abstract

**Graphical abstract:**
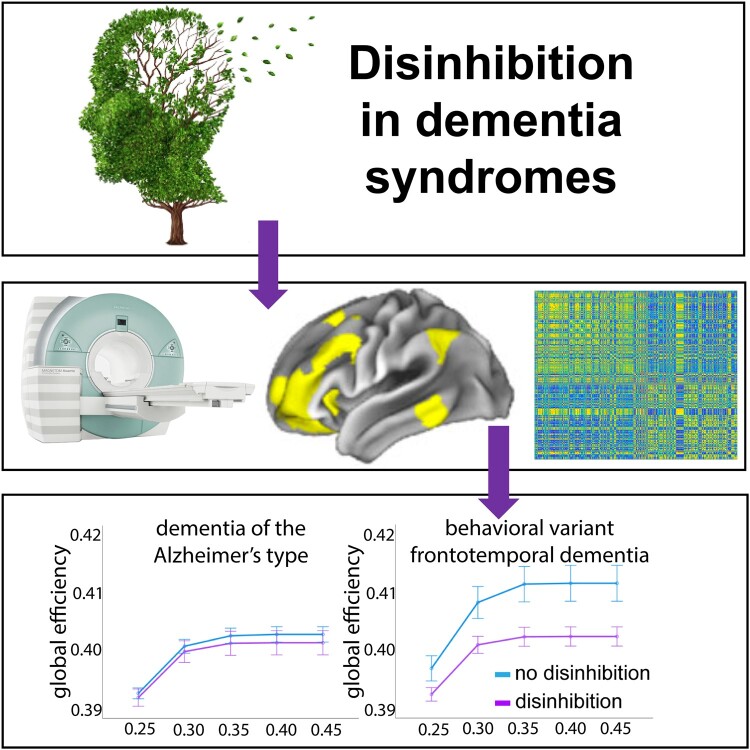

## Introduction

Disinhibition is one of the most distressing neuropsychiatric symptoms of dementia,^1,2^ involving socially inappropriate behaviours, such as hypersexual comments, excessive jocularity and inappropriate approaching of strangers.^3^ Disinhibition increases risk for institutionalization,^4^ and is associated with several worse clinical outcomes, including dementia severity, memory and executive functioning scores, as well as younger age.^3^ Early disinhibition is a key criterion for a clinical diagnosis of behavioural variant frontotemporal dementia (bvFTD) and is present in around three quarters of individuals with bvFTD.^5^ Disinhibition is also present in other forms of dementia, including in dementia of the Alzheimer’s type (DAT), albeit at a lower prevalence of around 17–30%.^6,7^ Neuroanatomically, bvFTD and DAT tend to show distinct patterns of cortical atrophy involving different functional brain networks; however, atypical presentations of these atrophy patterns can occur.^8-10^ These unique atrophy patterns are associated with unique pathological mechanisms, such as TDP-43 within bvFTD and Alzheimer’s disease pathology within DAT; however, individuals often have combinations of dementia pathologies at autopsy.^11^ Studies suggest that neurodegeneration is intimately linked to behavioural syndromes such as disinhibition, regardless of aetiology.^12^ As such, a transdiagnostic understanding of common symptoms that cross conventional disease boundaries is warranted, as mechanistic explanations may not exist at the level of molecular pathology^13^ but likely do at the network level.^14^

Most existing studies exploring the relationship between neuropsychiatric symptoms such as disinhibition and brain changes in dementia have related symptoms to atrophy,^15^ atrophy patterns^16^ or white matter abnormalities such as fractional anisotropy.^15^ However, such an approach is limited because it ignores the natural functional organization of the brain into networks of interconnected regions that are more closely aligned to the functional impairment than isolated brain regions^17^ and that brain diseases are therefore fundamentally influenced by brain network organization.^18^ Existing neuroimaging studies of disinhibition in dementia have identified regions of atrophy including in the orbitofrontal cortex (OFC) and insula (see^19^ for a review). These regions are part of the salience network (SN), which is involved in signaling the personal significance of stimuli, whether emotional, homeostatic, or cognitive, that requires adjustment in sympathetic tone.^20^ Regions of another major large-scale functional brain network, the cognitive control network (CCN) have also been associated with disinhibition in dementia, including the inferior frontal gyrus and dorsolateral prefrontal cortex (see^19^ for review). The CCN (also known as the central executive network or the executive control network) is important for higher-level cognitive processes, including executive functions.^21^ These large-scale intrinsic brain networks can be studied using both functional or structural MRI.

A relatively new method to explore structural brain networks is to construct Morphometric Similarity Networks (MSNs).^22^ This method takes several topological features (e.g. grey matter volume, cortical thickness, intrinsic curvature) of brain structure for a parcellated set of anatomical regions, and correlates them with the set of topological features of all other anatomical regions to produce a structural connectome (matrix) of inter-regional similarity. MSNs have been shown to recapitulate known cytoarchitectonic divisions, reflect axonal connectivity derived from macaque tract-tracing studies, and to predict inter-individual differences in cognition.^22^ Thus, MSNs are a biologically plausible and robust method for measuring structural brain connectivity and integrity. In addition to examining the whole brain as a network, sub-networks can be identified using published functional atlas parcellations, and this is the approach taken in the present study.

We aimed to determine the association between disinhibition and two major properties of brain networks: their ability to perform specialized information processing within densely connected groups of brain regions (network segregation) and their ability to rapidly combine specialized information from distributed brain regions (network integration).^23^ A handful of existing studies in individuals with DAT (but not bvFTD) have measured structural brain networks and identified alterations in segregation and integration. For example, Ferreria *et al*.^24^ found that measures of integration and segregation differ between those with distinct atrophy subtypes (e.g. limbic-predominant), not only with networks associated with their atrophy but also extending to other brain regions, and even in the minimal atrophy group. Palesi *et al*.^25^ also found aberrant alterations in brain network integration and segregation in individuals with DAT. Patterns of integration and segregation differed for different brain networks, and furthermore, these measures were related to cognitive performance.^25^ A lack of studies, however, have examined structural brain networks in relation to neuropsychiatric symptoms of dementia, such as disinhibition. In our literature review of neuroimaging studies of disinhibition across DAT and bvFTD,^19^ we found that brain regions frequently implicated included the orbitofrontal cortex and anterior cingulate cortex of the SN and the inferior frontal gyri and dorsolateral prefrontal cortex of the CCN. Based on our review, we presented a transdiagnostic theoretical model for disinhibition in dementia^19^ which posits that within the context of impaired cognitive control due to dementia, disinhibition is also associated with damage to the salience network. Thus, in the current study we analyzed morphometric similarity of the SN and CCN in DAT and bvFTD, leveraging large consortia neuroimaging datasets—the Alzheimer’s Disease Neuroimaging Initiative (ADNI,^26^) and the Frontotemporal Lobar Degeneration Neuroimaging Initiative (FTLDNI,^27^)—as well as data collected at Northwestern University’s Mesulam Center for Cognitive Neurology and Alzheimer’s disease. We hypothesized that individuals with disinhibition would have reduced network integrity (as measured by aberrant network segregation and integration) in the salience and CCNs compared to individuals without disinhibition, regardless of a clinical DAT or bvFTD diagnosis.

## Materials and methods

### Participants

Data from a total of 186 research participants were included in the study, shared from three sources: the Mesulam Center for Cognitive Neurology and Alzheimer's Disease (MCCNAD) at Northwestern University, the Alzheimer’s Disease Neuroimaging Initiative (ADNI), and the Frontotemporal Lobar Degeneration Neuroimaging Initiative (FTLDNI). Participants were included if they had i) a clinical diagnosis of DAT or bvFTD, ii) a T1-weighted MRI and iii) data from the Neuropsychiatric Inventory Questionnaire (NPI-Q, see below).

ADNI MRI data were from participants at the month 6 visit in the ADNI-2 protocol. Due to low numbers of DAT participants with moderate or severe disinhibition, from other visits (for which MRI and NPI-Q were within 6 months of each other) we added another five DAT participants with moderate disinhibition and two with severe disinhibition. ADNI was launched in 2003 as a public–private partnership, led by Principal Investigator Michael W. Weiner, MD. The primary goal of ADNI has been to test whether serial magnetic resonance imaging (MRI), positron emission tomography, other biological markers, and clinical and neuropsychological assessment can be combined to measure the progression of mild cognitive impairment (MCI) and early dementia due to Alzheimer's disease (AD). For up-to-date information, see www.adni-info.org. FTLDNI was begun in 2010 to identify neuroimaging modalities and methods of analysis for tracking frontotemporal lobar degeneration (FTLD) and to assess the value of imaging versus other biomarkers in diagnostic roles. The data were the result of collaborative efforts at three sites in North America. For information on participation and protocol, please visit http://memory.ucsf.edu/research/studies/nifd. Participants from the Mesulam Center cohort were enrolled in the Clinical and Imaging Cores of the Northwestern Alzheimer’s Disease Center funded by the National Institute on Aging.

Individuals were clinically diagnosed as either bvFTD or DAT based on clinical consensus without autopsy confirmation. Demographic and clinical details are shown in Table 1. The bvFTD group was significantly more likely to have disinhibition and had greater severity of disinhibition than the DAT group. There were also expected diagnostic differences in age and Clinical Dementia Rating Scale Sum of Boxes (CDR-SB), both of which were covaried in all analyses.

**Table 1:** Demographic and clinical characteristics

	DAT (n = 111)	bvFTD (n = 75)	Statistic
Age years	74.15 (7.53)	64.46 (7.03)	*t*(184) = 8.84, *P* < 0.001
Sex M/F	68/43	51/24	χ^2^(1) = 0.88, *P* = 0.348
White/Black/Asian/multiple/unknown	105/2/2/2/0	63/9/0/2/1	Fisher’s exact = 10.80, *P* = 0.007
Education years	15.24 (3.31)	16.05 (3.10)	*t*(184) = −1.67, *P* = 0.095
CDR-SB	5.38 (2.58)	8.16 (3.94)	*t*(114.64) = −5.33, *P* < 0.001
Days MRI—NPI-Q	14.01 (55.81)	4.03 (25.47)	*t*(165.04) = 2.96, *P* = 0.004
Database ADNI/FTLDNI/Mesulam	101/0/10	0/63/12	
Disinhibition Yes/No	27/84	62/13	χ^2^(1) = 61.05, *P* < 0.001
Disinhibition Severity 0/1/2/3	84/12/13/2	13/15/27/20	χ^2^(3) = 67.49, *P* < 0.001
Scanner Siemens/GE/Philips	59/32/20	61/14/0	χ^2^(1) = 20.89, *P* < 0.001

M, male; F, female; CDR-SB, Clinical Dementia Rating Scale Sum of Boxes; NPI-Q, Neuropsychiatric Inventory Questionnaire; ADNI, Alzheimer’s Disease Neuroimaging Initiative; FTLDNI, Frontotemporal Lobar Degeneration Neuroimaging Initiative; Mesulam, Mesulam Center for Cognitive Neurology and Alzheimer’s Disease; GE, General Electric.

### Measures

**Figure 1:**
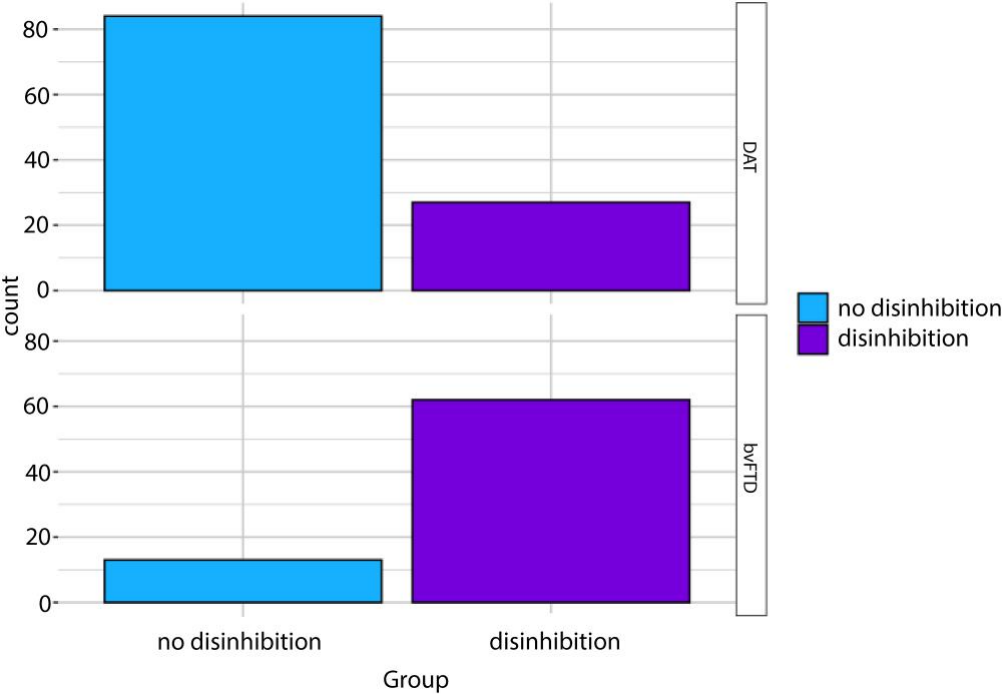
Disinhibition presence by diagnosis.

The Neuropsychiatric Inventory Questionnaire (NPI-Q)^28^ is a caregiver-based behavioural symptom assessment for individuals with cognitive impairment and dementia. It surveys 12 global behaviours over the previous month on their presence (yes/no) and severity (1 = mild, 2 = moderate, 3 = severe). One item per behaviour measures delusions, hallucinations, agitation/aggression, depression, anxiety, elation/euphoria, apathy, disinhibition, irritability/lability, aberrant motor behaviour, sleep disturbances and appetite/eating disturbances. The *disinhibition* item includes prompts like talking familiarly to strangers, acting impulsively, and saying things that are hurtful to others. Figure 1 shows the distribution of NPI-Q caregiver ratings of disinhibition presence (i.e. a severity rating of 1, 2 or 3) by diagnosis.

### MRI acquisition and processing

Only T1-weighted (T1w) MRI data acquired at 3T were included in this study. FTLDNI data were acquired at two sites, the University of California San Francisco (UCSF) and the Mayo clinic. At UCSF, a Siemens Tim Trio scanner acquired a volumetric magnetization-prepared rapid gradient echo (MPRAGE) sequence with the following parameters: slice orientation = coronal, slice thickness = 1mm, in-plane resolution = 1mm × 1mm, matrix = 240 × 256, repetition time (TR) = 2.3ms, echo time (TE) = 3ms, inversion time (TI) = 900ms, flip angle = 9°. At Mayo clinic, a General Electric (GE) Discovery (MR750) scanner acquired structural T1w images using the following parameters: slice orientation = coronal, slice thickness = 1.2mm, in-plane resolution = 1.0156 mm × 1.0156 mm, matrix = 256 × 256, TR = 7.3 ms, TE = 3 ms, TI = 900 ms, flip angle = 8°. The ADNI-2 protocol uses a MPRAGE with the following acquisition parameters: slice orientation = sagittal, slice thickness = 1.2 mm, TR = 2400 ms, TI = 1000 ms. ADNI-2 data are collected on a combination of Siemens, GE and Phillips scanners (see Table 1). For additional details, see (https://adni.loni.usc.edu/methods/documents/mri-protocols/). T1w MRIs from the Mesulam Center cohort were collected on Siemens Trio (ADNI-2 protocol, see above) and Prisma scanners. Prisma scanners use the advanced ADNI-3 protocol which collects an accelerated MPRAGE with the following parameters: orientation = sagittal, slice thickness = 1 mm, TR = 2300 ms, TE = 2.98 ms, TI = 900 ms, flip angle = 9°.

For each individual, T1w images were processed through FreeSurfer image analysis suite to generate cortical surfaces (version 6.0;^29^ see http://surfer.nmr.mgh.harvard.edu/). A total of 223 T1w images were processed with FreeSurfer (127 DAT from ADNI, 68 bvFTD from NIFD, 11 DAT and 17 bvFTD from MCCNAD). Manual edits were made when necessary using Freeview software. This included fixing over-segmentation (e.g. when it incorporated the dura) or under-segmentation of the grey matter after processing, then re-running Freesurfer with the adjusted marker to account for over- or under-segmentation. Of the 37 subjects excluded, 3 were excluded due to a processing error, 1 was excluded due to under-segmentation, and 33 were excluded due to over-segmentation. A total of 186 participants passed visual quality assurance and were included in the analysis (see Table 1).

Cortical surfaces were further parcellated into 360 regions of interest (ROIs) according to a validated atlas from the Human Connectome Project.^30^ Then, using an atlas based on functional network classifications,^31^ we identified ROIs within the salience network (SN) and the CCN. We defined the SN as comprising 56 parcels of the Cingulo-opercular and 6 parcels of the Orbito-affective network, and the CCN as comprising 23 parcels of the Dorsal attention and 50 parcels of the Frontoparietal network.^31^

### Specific network analysis

Morphometric similarity networks (MSNs) were originally proposed using multimodal data, including T1-weighted, diffusion and magnetization transfer (MT) images.^22^ However, diffusion and MT data do not exist for this sample. The use of single-modality, T1-weighted only MSNs has been shown to produce connectomes that are similar to complex connectomes,^32^ therefore the current analysis was restricted to grey matter networks. To generate individual MSNs, for each of the 360 surface ROIs, we utilized the following 7 surface-based cortical grey matter metrics provided by FreeSurfer processing: grey matter volume (GM), surface area (SA), cortical thickness (CT), intrinsic curvature (IC), mean curvature (MC), curved index (CI), and folding index (FI). Pairwise inter-regional Pearson correlations of morphometric feature vectors were calculated to produce a 360 × 360 morphometric similarity matrix for each individual. Self- and negative correlations were removed. Connectomes (including MSNs) are often thresholded to remove weak or spurious connections, as they may obscure the topology of strong and significant connections.^23^ Threshold values are often arbitrarily determined, thus ideally networks should be characterized across a broad range of thresholds^23^ and this is the approach that we, and others (e.g.^32^) have taken. We performed density thresholding on the weighted, undirected MSNs at 0.25, 0.30, 0.35, 0.40 and 0.45. These thresholds were chosen as thresholds below 0.25 resulted in disconnected networks. Optimal network architecture involves both functionally segregated (specialized) modules and a healthy number of integrated (inter-modular) links.^23^ This is referred to as small-world connectivity. In our data, SN small worldness in the DAT group ranged from 1.66 (SD = 0.16) at the 0.25 threshold to 1.12 (SD = 0.07) at the 0.45 threshold. SN small worldness in the bvFTD group ranged from 1.63 (SD = 0.19) to 1.12 (SD = 0.06). CCN small worldness in the DAT group ranged from 1.68 (SD = 0.14) at the 0.25 threshold to 1.14 (SD = 0.04) at the 0.45 threshold, and for bvFTD CCN small worldedness ranged from 1.68 (SD = 0.11) at the 0.25 threshold to 1.14 (SD = 0.04) at the 0.45 threshold. Finally, each weighted MSN was normalized.

Graph theoretical metrics for each MSN were calculated using Brain Connectivity Toolbox^23^ implemented in MATLAB. Two metrics were calculated in the current analysis. *Transitivity* is the normalized clustering coefficient, normalized across the whole network to reduce sensitivity due to poorly connected nodes in a graph. As a measure of segregation, transitivity reflects how much nodes cluster together. The second measure was *Global efficiency*, which is defined as the average of the inverse of the shortest path length over the network. It is a measure of network integration.^23^

### Statistical analysis

For each MSN metric within each network, a 2 (disinhibition presence) × 2 (diagnosis) × 5 (threshold) repeated measures ANCOVA was conducted. We tested for the (between-subjects) main effects of diagnosis, disinhibition presence, and their interaction. Threshold was a within-subjects factor. This was included to ensure that the results were not threshold-dependent, as there is no agreed-upon threshold for MSNs in the literature. This resulted in a total of 4 statistical results: network transitivity within the SN, global efficiency within the SN, network transitivity within the CCN, and global efficiency within the CCN. Covariates in all models were age, sex, total intracranial volume, days between MRI and NPI-Q, dementia severity (CDR-SB), scanner type (dummy coded with Siemens as the reference group), and education (years). Full models are reported in Supplemental Tables 1–4. Sensitivity analyses additionally covarying race are reported in Supplemental Tables 5–8.

## Results

### Salience network transitivity

**Figure 2:**
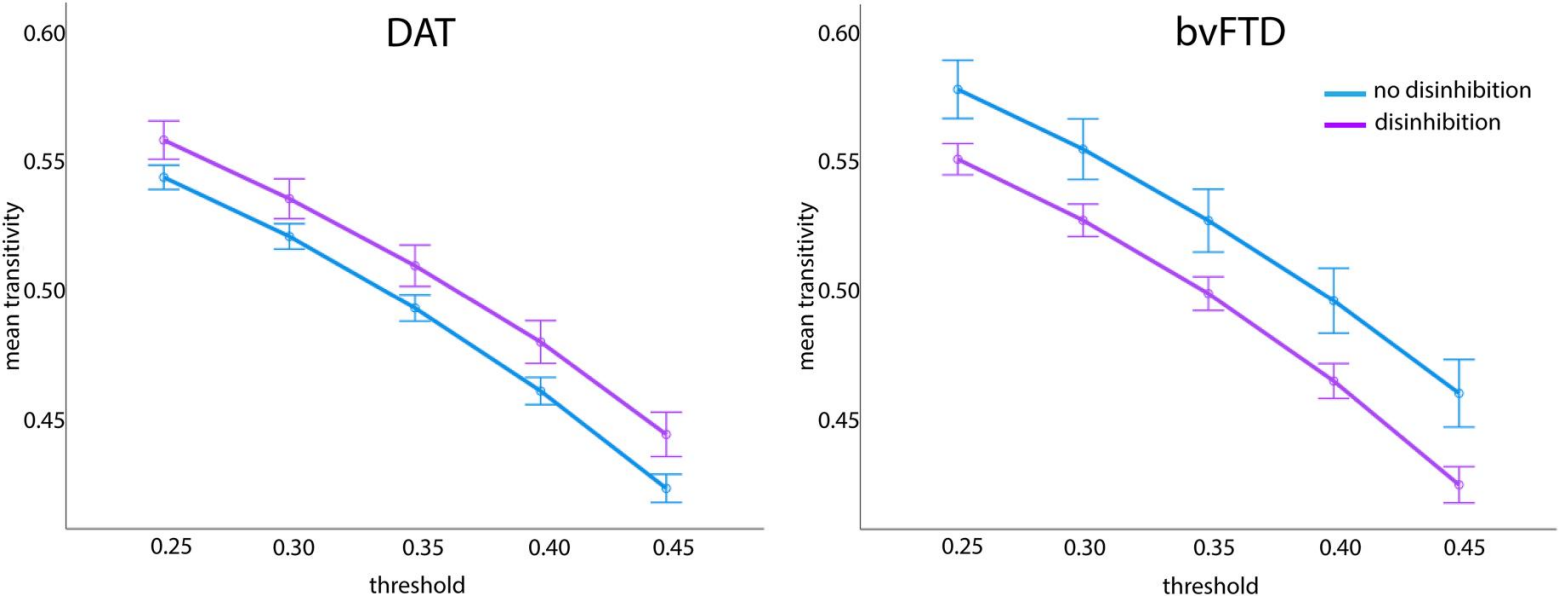
**Results of a 2 (disinhibition presence) × 2 (diagnosis) × 5 (threshold) repeated measures ANCOVA for salience network transitivity.** A significant diagnosis by presence of disinhibition interaction was observed, F(1, 172) = 8.92, *P* = 0.003, η_p_2 = 0.05.

There was a significant interaction between diagnosis and presence of disinhibition for transitivity of the salience network, F(1, 172) = 8.92, *P* = 0.003, η_p_2 = 0.05. Figure 2 shows that individuals with DAT with disinhibition had higher SN transitivity than individuals with DAT without disinhibition, whereas individuals with bvFTD and disinhibition had lower SN transitivity than individuals with bvFTD without disinhibition.

### Salience network global efficiency

**Figure 3:**
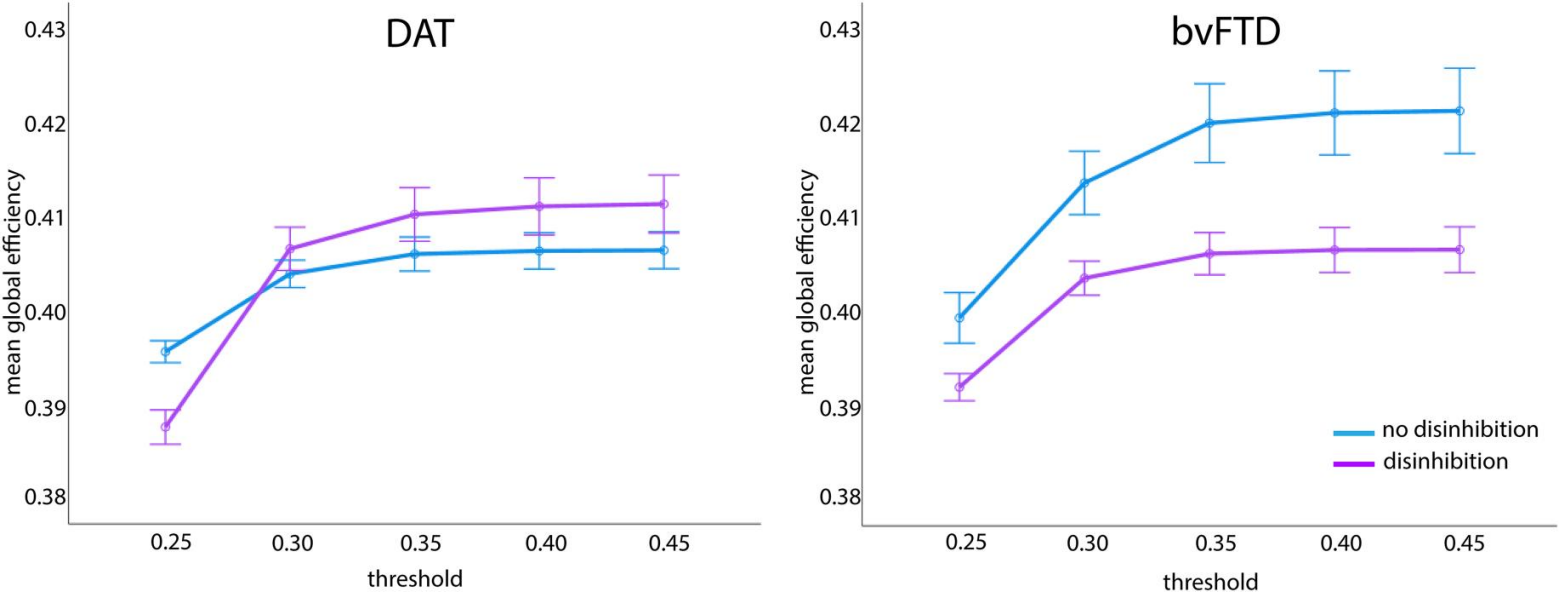
**Results of a 2 (disinhibition presence) × 2 (diagnosis) × 5 (threshold) repeated measures ANCOVA for salience network global efficiency.** A significant diagnosis by presence of disinhibition interaction was observed, F(1, 172) = 8.06, *P* = 0.005, η_p_2 = 0.05.

There was a significant interaction between diagnosis and presence of disinhibition for global efficiency of the salience network, F(1, 172) = 8.06, *P* = 0.005, η_p_2 = 0.05. Figure 3 shows that individuals with DAT with disinhibition had higher SN global efficiency than individuals with DAT without disinhibition, whereas individuals with bvFTD with disinhibition had lower SN global efficiency that individuals with bvFTD without disinhibition.

### Cognitive control network transitivity

**Figure 4:**
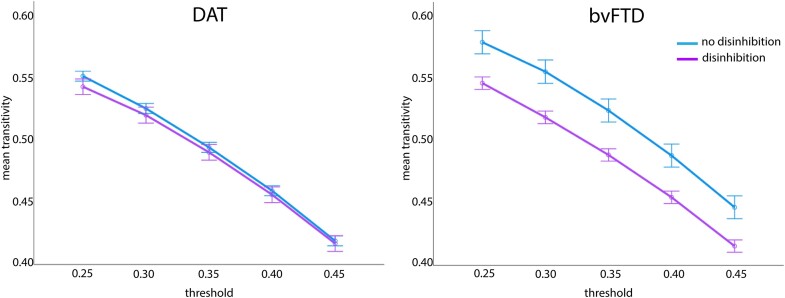
**Results of a 2 (disinhibition presence) × 2 (diagnosis) × 5 (threshold) repeated measures ANCOVA for cognitive control network transitivity.** A significant diagnosis by presence of disinhibition was observed, F(1, 172) = 5.90, *P* = 0.016, η_p_2 = 0.03.

There was a significant interaction between diagnosis and presence of disinhibition for transitivity of the CCN, F(1, 172) = 5.90, *P* = 0.016, η_p_2 = 0.03. Figure 4 shows that DAT with disinhibition did not differ in CCN transitivity from DAT without disinhibition, whereas bvFTD with disinhibition showed lower transitivity than bvFTD without disinhibition.

### Cognitive control network global efficiency

**Figure 5:**
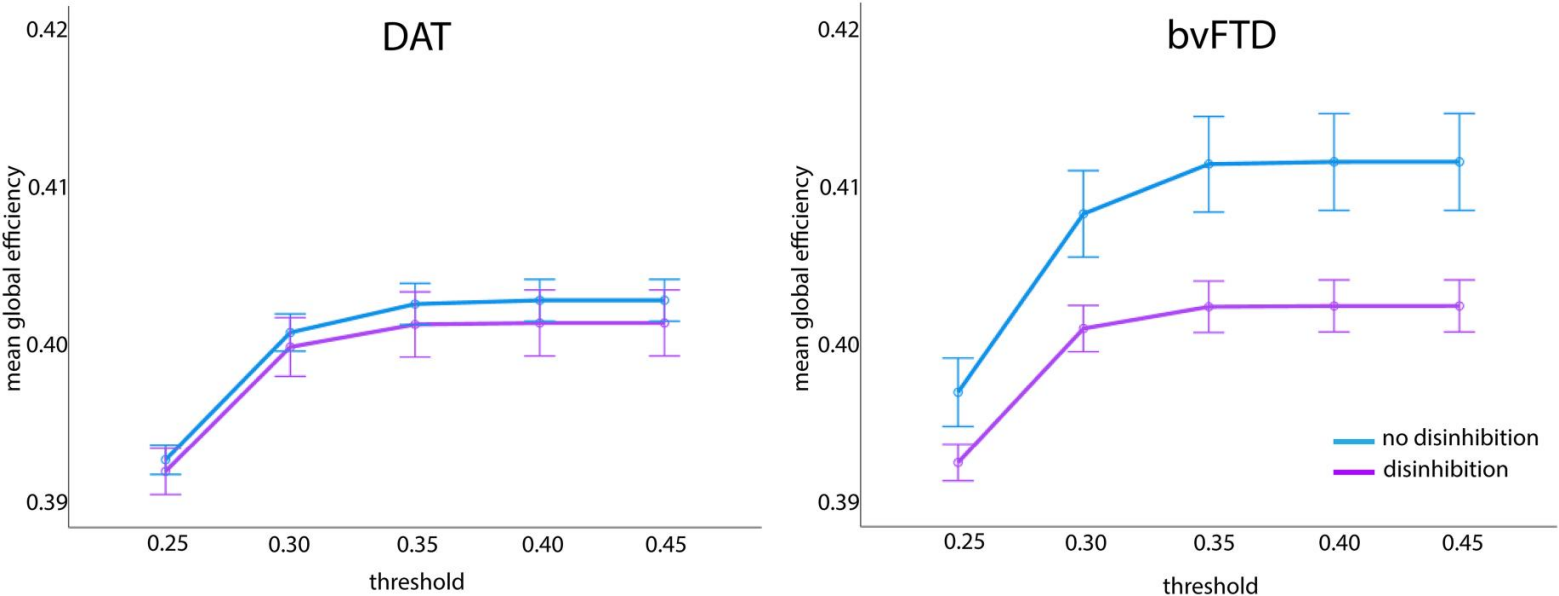
**Results of a 2 (disinhibition presence) × 2 (diagnosis) × 5 (threshold) repeated measures ANCOVA for cognitive control network global efficiency.** There was a significant main effect of disinhibition presence, F(1, 172) = 5.95, *P* = 0.016, η_p_2 = 0.03. In both DAT and bvFTD, individuals with disinhibition had lower CCN global efficiency than those without disinhibition.

Global efficiency of the CCN differed significantly between those with versus without disinhibition, F(1, 172) = 5.95, *P* = 0.016, η_p_2 = 0.03. Figure 5 shows that both DAT and bvFTD with disinhibition had lower CCN global efficiency than DAT and bvFTD without disinhibition. Global efficiency of the CCN was not due to an interaction between diagnosis and disinhibition, F(1, 172) = 3.16, *P* = 0.077, η_p_2 = 0.02. There was also no significant difference in global efficiency of the CCN between DAT and bvFTD, F(1, 172) = 3.69, *P* = 0.056, η_p_2 = 0.02.

## Discussion

This is the first study to examine morphometric similarity networks (MSNs) as they relate to neuropsychiatric symptoms in dementia. We focused on two clinical syndromes of dementia that both experience disinhibition, albeit in different prevalence and severity, to determine whether alterations in transitivity and global efficiency of the salience (SN) and cognitive control (CCN) networks associated with disinhibition were common across clinical diagnoses. It should be emphasized that we did not have in vivo or post mortem pathological validation so that this study focuses on behavioural expressions and symptoms regardless of their biological substrates. In partial support of our hypothesis, we found that disinhibition presence was associated with reduced global efficiency of the CCN, regardless of diagnosis. We therefore suggest that global efficiency of the CCN could be an underlying mechanism of disinhibition that is shared across bvFTD and DAT clinical syndromes.

We anticipated significant associations between disinhibition and transitivity, as reduced whole-brain transitivity has been reported in DAT compared to controls using a method related to MSNs known as structural covariance networks.^33^ Furthermore, given previous findings in dementia implicating regions of the salience network in disinhibition (see^19^ for review) and measures of social functioning such as interpersonal warmth, socioemotional sensitivity, and empathy,^34-36^ we predicted that reduced integrity of the SN (measured using transitivity and global efficiency) would be associated with disinhibition in our sample. However, contrary to our hypothesis, our analyses of transitivity of the CCN and SN and of global efficiency of the SN revealed significant interactions between presence of disinhibition and clinical diagnosis. Individuals with bvFTD, who are more likely to have disinhibition than DAT, have previously been found to show reduced connectivity of the SN compared to individuals with DAT, who in turn showed increased connectivity of the SN (and decreased connectivity of the default mode network, in contrast to bvFTD).^37^ These connectivity profiles are consistent with diagnostic differences in brain atrophy between DAT and bvFTD.^38^ These previous studies suggested that the observed patterns reflect the strength and deficit profiles of the two diagnoses. In the present study, however, we explicitly tested the association between network changes and the symptom of disinhibition, across diagnoses. Thus, although bvFTD and DAT show diagnostic differences in brain networks, the symptom of disinhibition, which occurs at least to some extent in both diagnoses, shares a common underlying neural substrate, that of decreased global efficiency of the CCN.

A small number of studies have now investigated brain networks using graph theory or connectome modelling in dementia;^39-41^ however, no studies have used MSNs to identify associations with neuropsychiatric symptoms. Some have applied graph theory to resting state fMRI data to examine associations with neuropsychiatric symptoms, e.g. Golbabaei *et al*.^42^ and Ng *et al*.,^43^ however these studies only examined NPI-Q total scores, not disinhibition specifically. Reyes *et al*.^44^ measured behavioural changes in FTLD clinical variants using the Frontal System Behaviour Scale (FrSBe) Total and Inhibition scores, and found that increased whole-brain global efficiency was related to more severe deficits on the FrsBe. These results are in the opposing direction to our findings; however, Reyes *et al*. measured resting state functional connectivity, which is often associated with increases that are interpreted to be compensatory. A study of cognitively normal individuals from the Human Connectome Project (HCP)^45^ used MSNs to study inhibitory control using the flanker task. Using connectome predictive modelling they found that inhibitory control ability was predicted by morphometric similarity in several prefrontal regions, in particular the OFC and inferior frontal gyrus. These HCP results, combined with the present findings, indicate that morphometric similarity, including of prefrontal regions, can predict inhibitory control.

Several existing studies have implicated non-frontal regions in disinhibition in dementia syndromes. For example, Zamboni *et al*.^46^ found disinhibition severity correlated with atrophy in the right superior temporal sulcus, right mediotemporal limbic structures and right nucleus accumbens. Santillo *et al*.^15^ found disinhibition was associated with reduced cortical thickness in the right insula and parahippocampal gyrus. They (and others^47^) also found that disinhibition was associated with reduced integrity of the uncinate fasciculus, which connects the anterior temporal lobe to orbitofrontal cortex. These studies, together with the results presented here, call into question the conceptualization of disinhibition as a ‘prefrontal’ syndrome.^2,15^ Similar to our theoretical model that posits that the most severe disinhibition is observed when there is either damage to both the ventromedial prefrontal cortex (VMPFC), including OFC, and the amygdala or a disconnection of the VMPFC and amygdala, others have suggested a loss of control of limbic system by the frontal system that results in disinhibition,^46^ and that the breakdown of connections between the temporal lobe and OFC is important for disinhibition, rather than isolated loss of specific functions.^2^ Consistent with this notion, in a transdiagnostic study of individuals with dementia, including frontotemporal dementia subtypes and DAT, Multani *et al*.^48^ found that resting state fMRI of the left inferior temporal gyrus anterior division to the bilateral frontal pole and paracingulate gyrus was positively associated with social cognition. Other studies have found frontoparietal connectivity changes to be associated with behavioural impairment in individuals at risk for dementia. Matsuoka *et al*.^49^ examined resting state fMRI in individuals with amnestic MCI and found that lower frontoparietal control network connectivity was associated with more severe Total and affective dysregulation score on the Mild Behavioural Impairment checklist (MBI-C). This finding supports our results as the CCN in the present study was comprised of the frontoparietal and dorsal attention networks.^31^ Thus, dysconnectivity of widely distributed brain regions is posited and has been shown in previous research to be associated with disinhibition. The main finding of the present study that reduced global efficiency of the CCN is associated with presence of disinhibition in both DAT and bvFTD supports previous findings that disinhibition is associated with reduced connectivity of distributed brain regions and indicates that disinhibition is associated with reduced integration of information from distant brain regions implicated in cognitive control.

## Limitations

This study has several limitations. First, MSNs are only a proxy for anatomical connectivity, as they measure the similarity of pairs of cortical regions based on the seven morphometric features. High similarity between regions could indicate shared contribution by heredity when organizing groups of brain areas that develop together in evolution, or they could also reflect experience-related plasticity in a set of brain regions.^45^ However, MSNs have been found to be robust, capture functional, cellular, and molecular features of the brain, and predict inter-individual differences in cognition.^22^ MSNs capture known cortical cytoarchitecture and axonal connectivity, and grey matter networks have been shown to overlap with functional brain networks.^50^ Another limitation is the group difference in age, as bvFTD has a much earlier mean age of onset than DAT. We attempted to account for this by covarying age in all analyses. There was also a lack of racial diversity in our sample, limiting the generalizability of the findings. This limitation has been common in research on the biology of cognitive and emotional aging and requires ongoing emphasis on recruitment of under-represented communities. Another limitation is that we only examined cortical regions. In particular, the amygdala is a key node of the salience network. Results for the SN may have been different were we to have included the amygdala in this MSN, as, for example, atrophy of functional networks connected to the amygdala has been shown to relate to different types and severity of social impairments in FTLD.^51^ We chose to utilize the Ji *et al*.^31^ functional network assignment of the Glasser *et al*.^30^ atlas, however different atlas choices may have affected the results. Another limitation is the unequal proportions of disinhibition between bvFTD and DAT. Whilst reflective of prevalence, it is possible that the DAT group was underpowered to detect effects of disinhibition presence. The use of the NPI-Q is a strength on one hand as it is the most widely utilized, ‘gold-standard’ measure of neuropsychiatric symptoms in dementia, but also a limitation because it does not assess disinhibition comprehensively. Other researchers have examined disinhibition in bvFTD using multidimensional measures, including compulsivity and social disinhibition, and identified differential patterns of atrophy^51^ and white matter changes.^47^ The CDR-SB was also designed for DAT, not bvFTD, therefore this covariate may have been biased. Another potential limitation is that we relied on clinical diagnoses rather than autopsy- or biomarker-confirmed diagnoses. However, we argue that because we hypothesized that the effects of disinhibition would be transdiagnostic, this is less of a concern. Despite these limitations, this study has substantially improved our previously very limited understanding of the relationships between disinhibition and structural brain networks.

## Conclusions and future directions

In the first study to use MSNs to study disinhibition in dementia, we found that individuals with disinhibition had significantly lower global efficiency of the CCN than individuals without disinhibition, regardless of clinical dementia syndrome. This study included individuals with different underlying disease types in an attempt to identify a disease-independent network association, as individuals can have multiple neuropathologies at once, and there is also heterogeneity of clinical presentations. Future work could examine whether these findings extend to disinhibition in other forms of dementia associated with neurodegenerative diseases, such as Parkinson’s^52^ and Huntington’s disease.^53^ rsfMRI data is not available in the FTLDNI dataset, but future research should investigate whether the structural network findings reported here can be replicated using resting state fMRI. This would be an important step on the path to identifying the potential for intervention at the level of the CCN to target disinhibition. Overall, the results of the present study indicate reduced global efficiency of the CCN in individuals with disinhibition, regardless of a clinical diagnosis of bvFTD or DAT, suggesting that transdiagnostically, disinhibition in dementia may be characterized by reduced ability of the cognitive control network to rapidly and effectively transfer information from distributed brain regions.

## Supplementary Material

fcae124_Supplementary_Data

## Data Availability

Data from analyses in this paper were publicly available data obtained from ADNI, https://adni.loni.usc.edu/data-samples/access-data/#access_data and FTLDNI, https://ida.loni.usc.edu/collaboration/access/appLicense.jsp; jsessionid=00D275AA711EC90C98F818E4B362ECA6. Data from the MCCNAD are available via collaborative request process https://www.brain.northwestern.edu/scientists-students/collaborative-request.html.

## Appendix

Data used in preparation of this article were obtained from the Frontotemporal Lobar Degeneration Neuroimaging Initiative (FTLDNI) database. The investigators at NIFD/FTLDNI contributed to the design and implementation of FTLDNI and/or provided data, but did not participate in analysis or writing of this report (unless otherwise listed). The FTLDNI investigators included the following individuals:

Howard Rosen, Bradford C. Dickerson, Kimoko Domoto-Reilly, David Knopman, Bradley F. Boeve, Adam L. Boxer, John Kornak, Bruce L. Miller, William W. Seeley, Maria-Luisa Gorno-Tempini, Scott McGinnis and Maria Luisa Mandelli.

## References

[1] Massimo L, Powers C, Moore P, et al Neuroanatomy of apathy and disinhibition in frontotemporal lobar degeneration. Dement Geriatr Cogn Disord. 2009;27(1):96–104. 19158440 10.1159/000194658 PMC2820577

[2] Migliaccio R, Tanguy D, Bouzigues A, et al Cognitive and behavioural inhibition deficits in neurodegenerative dementias. Cortex. 2020;131:265–283. 32919754 10.1016/j.cortex.2020.08.001 PMC7416687

[3] Finger E, Zhang J, Dickerson B, Bureau Y, Masellis M; Alzheimer’s Disease Neuroimaging Initiative. Disinhibition in Alzheimer’s disease is associated with reduced right frontal pole cortical thickness. J Alzheimers Dis. 2017;60(3):1161–1170. 28984590 10.3233/JAD-170348

[4] Scarmeas N, Brandt J, Blacker D, et al Disruptive behavior as a predictor in Alzheimer disease. Arch Neurol. 2007;64(12):1755–1761. 18071039 10.1001/archneur.64.12.1755 PMC2690610

[5] Rascovsky K, Hodges JR, Knopman D, et al Sensitivity of revised diagnostic criteria for the behavioural variant of frontotemporal dementia. Brain. 2011;134:2456–2477. 21810890 10.1093/brain/awr179 PMC3170532

[6] Zhao QF, Tan L, Wang HF, et al The prevalence of neuropsychiatric symptoms in Alzheimer’s disease: Systematic review and meta-analysis. J Affect Disord. 2016;190:264–271. 26540080 10.1016/j.jad.2015.09.069

[7] Craig D, Mirakhur A, Hart DJ, McIlroy SP, Passmore AP. A cross-sectional study of neuropsychiatric symptoms in 435 patients with Alzheimer’s disease. Am J Geriatr Psychiatry. 2005;13(6):460–468. 15956265 10.1176/appi.ajgp.13.6.460

[8] Johnson JK, Head E, Kim R, Starr A, Cotman CW. Clinical and pathological evidence for a frontal variant of Alzheimer disease. Arch Neurol. 1999;56(10):1233–1239. 10520939 10.1001/archneur.56.10.1233

[9] Ossenkoppele R, Pijnenburg YA, Perry DC, et al The behavioural/dysexecutive variant of Alzheimer’s disease: Clinical, neuroimaging and pathological features. Brain. 2015;138(Pt 9):2732–2749. 26141491 10.1093/brain/awv191 PMC4623840

[10] Rohrer JD, Ridgway GR, Modat M, et al Distinct profiles of brain atrophy in frontotemporal lobar degeneration caused by progranulin and tau mutations. Neuroimage. 2010;53(3):1070–1076. 20045477 10.1016/j.neuroimage.2009.12.088 PMC2941039

[11] Kertesz A, McMonagle P, Blair M, Davidson W, Munoz DG. The evolution and pathology of frontotemporal dementia. Brain. 2005;128(Pt 9):1996–2005. 16033782 10.1093/brain/awh598

[12] Perry DC, Brown JA, Possin KL, et al Clinicopathological correlations in behavioural variant frontotemporal dementia. Brain. 2017;140:3329–3345. 29053860 10.1093/brain/awx254 PMC5841140

[13] Husain M . Transdiagnostic neurology: Neuropsychiatric symptoms in neurodegenerative diseases. Brain. 2017;140:1535–1536. 28549134 10.1093/brain/awx115

[14] van den Heuvel MP, Sporns O. A cross-disorder connectome landscape of brain dysconnectivity. Nat Rev Neurosci. 2019;20:435–446. 31127193 10.1038/s41583-019-0177-6 PMC8864539

[15] Santillo AF, Lundblad K, Nilsson M, et al Grey and white matter clinico-anatomical correlates of disinhibition in neurodegenerative disease. PLoS One. 2016;11(10):e0164122. 27723823 10.1371/journal.pone.0164122 PMC5056728

[16] Ranasinghe KG, Rankin KP, Pressman PS, et al Distinct subtypes of behavioral variant frontotemporal dementia based on patterns of network degeneration. JAMA Neurol. 2016;73(9):1078–1088. 27429218 10.1001/jamaneurol.2016.2016 PMC5024785

[17] Wen W, He Y, Sachdev P. Structural brain networks and neuropsychiatric disorders. Curr Opin Psychiatr. 2011;24(3):219–225. 10.1097/YCO.0b013e32834591f8 21430538

[18] Fornito A, Zalesky A, Breakspear M. The connectomics of brain disorders. Nat Rev Neurosci. 2015;16(3):159–172. 25697159 10.1038/nrn3901

[19] Jenkins LM, Wang L, Rosen H, Weintraub S. A transdiagnostic review of neuroimaging studies of apathy and disinhibition in dementia. Brain. 2022;145(6):1886–1905. 35388419 10.1093/brain/awac133 PMC9630876

[20] Seeley WW, Menon V, Schatzberg AF, et al Dissociable intrinsic connectivity networks for salience processing and executive control. J Neurosci. 2007;27(9):2349–2356. 17329432 10.1523/JNEUROSCI.5587-06.2007 PMC2680293

[21] Menon V . Large-scale brain networks and psychopathology: A unifying triple network model. Trends Cogn Sci. 2011;15(10):483–506. 21908230 10.1016/j.tics.2011.08.003

[22] Seidlitz J, Vasa F, Shinn M, et al Morphometric similarity networks detect microscale cortical organization and predict inter-individual cognitive variation. Neuron. 2018;97(1):231–247.e7. 29276055 10.1016/j.neuron.2017.11.039 PMC5763517

[23] Rubinov M, Sporns O. Complex network measures of brain connectivity: Uses and interpretations. Neuroimage. 2010;52(3):1059–1069. 19819337 10.1016/j.neuroimage.2009.10.003

[24] Ferreira D, Pereira JB, Volpe G, Westman E; Alzheimer’s Disease Neuroimaging Initiative. Subtypes of Alzheimer’s disease display distinct network abnormalities extending beyond their pattern of brain atrophy. Front Neurol. 2019;10(13):524. 31191430 10.3389/fneur.2019.00524 PMC6547836

[25] Palesi F, Castellazzi G, Casiraghi L, et al Exploring patterns of alteration in Alzheimer’s disease brain networks: A combined structural and functional connectomics analysis. Front Neurosci. 2016;10:380. 27656119 10.3389/fnins.2016.00380 PMC5013043

[26] Weiner MW, Aisen PS, Jack CR, et al The Alzheimer’s disease neuroimaging initiative: Progress report and future plans. Alzheimers Dement. 2010;6(3):202–211. 20451868 10.1016/j.jalz.2010.03.007 PMC2927112

[27] Perry DC, Sturm VE, Seeley WW, Miller BL, Kramer JH, Rosen HJ. Anatomical correlates of reward-seeking behaviours in behavioural variant frontotemporal dementia. Brain. 2014;137:1621–1626. 24740987 10.1093/brain/awu075 PMC4032100

[28] Kaufer DI, Cummings JL, Ketchel P, et al Validation of the NPI-Q, a brief clinical form of the neuropsychiatric inventory. J Neuropsychiatr Clin Neurosci. 2000;12(2):233–239. 10.1176/jnp.12.2.233 11001602

[29] Fischl B . FreeSurfer. Neuroimage. 2012;62(2):774–781. 22248573 10.1016/j.neuroimage.2012.01.021 PMC3685476

[30] Glasser MF, Coalson TS, Robinson EC, et al A multi-modal parcellation of human cerebral cortex. Nature. 2016;536(7615):171–178. 27437579 10.1038/nature18933 PMC4990127

[31] Ji JL, Spronk M, Kulkarni K, Repovs G, Anticevic A, Cole MW. Mapping the human brain’s cortical-subcortical functional network organization. Neuroimage. 2019;185:35–57. 30291974 10.1016/j.neuroimage.2018.10.006 PMC6289683

[32] King DJ, Wood AG. Clinically-feasible brain morphometric similarity network construction approaches with restricted MRI acquisitions. Network Neuroscience. 2019;4(1):274–291. 10.1162/netn_a_00123 PMC7069065 32181419

[33] Pereira JB, Mijalkov M, Kakaei E, et al Disrupted network topology in patients with stable and progressive mild cognitive impairment and Alzheimer’s disease. Cereb Cortex. 2016;26(8):3476–3493. 27178195 10.1093/cercor/bhw128 PMC4961019

[34] Toller G, Brown J, Sollberger M, et al Individual differences in socioemotional sensitivity are an index of salience network function. Cortex. 2018;103:211–223. 29656245 10.1016/j.cortex.2018.02.012 PMC6143366

[35] Toller G, Yang WFZ, Brown JA, et al Divergent patterns of loss of interpersonal warmth in frontotemporal dementia syndromes are predicted by altered intrinsic network connectivity. NeuroImage Clinical. 2019;22:101729. 30836325 10.1016/j.nicl.2019.101729 PMC6403437

[36] Pasquini L, Nana AL, Toller G, et al Salience network atrophy links neuron type-specific pathobiology to loss of empathy in frontotemporal dementia. Cereb Cortex. 2020;30(10):5387–5399. 32500143 10.1093/cercor/bhaa119 PMC7566683

[37] Zhou J, Greicius MD, Gennatas ED, et al Divergent network connectivity changes in behavioural variant frontotemporal dementia and Alzheimer’s disease. Brain. 2010;133:1352–1367. 20410145 10.1093/brain/awq075 PMC2912696

[38] Seeley WW, Crawford RK, Zhou J, Miller BL, Greicius MD. Neurodegenerative diseases target large-scale human brain networks. Neuron. 2009;62(1):42–52. 19376066 10.1016/j.neuron.2009.03.024 PMC2691647

[39] He Y, Chen Z, Evans A. Structural insights into aberrant topological patterns of large-scale cortical networks in Alzheimer’s disease. J Neurosci. 2008;28(18):4756–4766. 18448652 10.1523/JNEUROSCI.0141-08.2008 PMC6670444

[40] Zheng W, Yao Z, Xie Y, Fan J, Hu B. Identification of Alzheimer’s disease and mild cognitive impairment using networks constructed based on multiple morphological brain features. Biol Psychiatry Cogn Neurosci Neuroimaging. 2018;3(10):887–897. 30077576 10.1016/j.bpsc.2018.06.004

[41] Voevodskaya O, Pereira JB, Volpe G, et al Altered structural network organization in cognitively normal individuals with amyloid pathology. Neurobiol Aging. 2018;64:15–24. 29316528 10.1016/j.neurobiolaging.2017.11.014

[42] Golbabaei S, Dadashi A, Soltanian-Zadeh H. Measures of the brain functional network that correlate with Alzheimer’s neuropsychological test scores: An fMRI and graph analysis study. Annu Int Conf IEEE Eng Med Biol Soc. 2016;2016:5554–5557. 28269515 10.1109/EMBC.2016.7591985

[43] Ng ASL, Wang J, Ng KK, et al Distinct network topology in Alzheimer’s disease and behavioral variant frontotemporal dementia. Alzheimers Res Ther. 2021;13(1):13. 33407913 10.1186/s13195-020-00752-w PMC7786961

[44] Reyes P, Ortega-Merchan MP, Rueda A, et al Functional connectivity changes in behavioral, semantic, and nonfluent variants of frontotemporal dementia. Behav Neurol. 2018;10:9684129. 10.1155/2018/9684129 PMC5902123 29808100

[45] He N, Rolls ET, Zhao W, Guo S. Predicting human inhibitory control from brain structural MRI. Brain Imaging Behav. 2020;14(6):2148–2158. 31346962 10.1007/s11682-019-00166-9

[46] Zamboni G, Huey ED, Krueger F, Nichelli PF, Grafman J. Apathy and disinhibition in frontotemporal dementia: Insights into their neural correlates. Neurology. 2008;71(10):736–742. 18765649 10.1212/01.wnl.0000324920.96835.95 PMC2676948

[47] Tanguy D, Batrancourt B, Estudillo-Romero A, et al An ecological approach to identify distinct neural correlates of disinhibition in frontotemporal dementia. NeuroImage Clinical. 2022;35:103079. 35700600 10.1016/j.nicl.2022.103079 PMC9194654

[48] Multani N, Taghdiri F, Anor CJ, et al Association between social cognition changes and resting state functional connectivity in frontotemporal dementia, Alzheimer’s disease, Parkinson’s disease, and healthy controls. Front Neurosci. 2019;13:1259. 31824254 10.3389/fnins.2019.01259 PMC6883726

[49] Matsuoka T, Ueno D, Ismail Z, et al Neural correlates of mild behavioral impairment: A functional brain connectivity study using resting-state functional magnetic resonance imaging. J Alzheimers Dis. 2021;83(3):1221–1231. 34420972 10.3233/JAD-210628 PMC8543254

[50] Zhou J, Seeley WW. Network dysfunction in Alzheimer’s disease and frontotemporal dementia: Implications for psychiatry. Biol Psychiatry. 2014;75(7):565–573. 24629669 10.1016/j.biopsych.2014.01.020

[51] Bickart KC, Brickhouse M, Negreira A, Sapolsky D, Barrett LF, Dickerson BC. Atrophy in distinct corticolimbic networks in frontotemporal dementia relates to social impairments measured using the social impairment rating scale. J Neurol Neurosurg Psychiatr. 2014;85(4):438–448. 10.1136/jnnp-2012-304656 PMC4315506 24133285

[52] Weintraub D, Koester J, Potenza MN, et al Impulse control disorders in Parkinson disease: A cross-sectional study of 3090 patients. Arch Neurol. 2010;67(5):589–595. 20457959 10.1001/archneurol.2010.65

[53] Paulsen JS, Ready RE, Hamilton JM, Mega MS, Cummings JL. Neuropsychiatric aspects of Huntington’s disease. J Neurol Neurosurg Psychiatr. 2001;71(3):310–314. 10.1136/jnnp.71.3.310 PMC1737562 11511702

