# Supplementary material for: Disinhibition in dementia related to reduced morphometric similarity of cognitive control network
Source: Brain Commun. 2024 Apr 16;6(2):fcae124. doi: 10.1093/braincomms/fcae124 (PMC11044061; doi:10.1093/braincomms/fcae124)
Supplement: fcae124_Supplementary_Data [file fcae124_supplementary_data.docx]

**Contents**

Supplementary Table 1. Results of Salience network transitivity 2 (diagnosis) x 2 (disinhibition presence) x 5 (threshold) repeated measures ANCOVA

Supplementary Table 2. Results of Salience network Global efficiency 2 (diagnosis) x 2 (disinhibition presence) x 5 (threshold) repeated measures ANCOVA

Supplementary Table 3. Results of Cognitive control network transitivity 2 (diagnosis) x 2 (disinhibition presence) x 5 (threshold) repeated measures ANCOVA

Supplementary Table 4. Results of Cognitive control network Global efficiency 2 (diagnosis) x 2 (disinhibition presence) x 5 (threshold) repeated measures ANCOVA

Supplementary Table 5. Results of Salience network transitivity 2 (diagnosis) x 2 (disinhibition presence) x 5 (threshold) repeated measures ANCOVA, with race additionally covaried

Supplementary Table 6. Results of Salience network Global efficiency 2 (diagnosis) x 2 (disinhibition presence) x 5 (threshold) repeated measures ANCOVA, with race additionally covaried

Supplementary Table 7. Results of Cognitive control network transitivity 2 (diagnosis) x 2 (disinhibition presence) x 5 (threshold) repeated measures ANCOVA, with race additionally covaried

Supplementary Table 8. Results of Cognitive control network Global efficiency 2 (diagnosis) x 2 (disinhibition presence) x 5 (threshold) repeated measures ANCOVA, with race additionally covaried

**Supplementary Table 1. Results of Salience Network Transitivity 2 (diagnosis) x 2 (disinhibition presence) x 5 (threshold) repeated measures ANCOVA**

| Source | Type III Sum of Squares | df | Mean Square | F | Sig. | Partial Eta Squared |
| --- | --- | --- | --- | --- | --- | --- |
| Intercept | 0.95 | 1 | 0.95 | 125.70 | <.001 | 0.42 |
| Estimated total ICV | <0.01 | 1 | <0.01 | 0.17 | .678 | 0.00 |
| Days between MRI and NPI-Q | <0.01 | 1 | <0.01 | 0.35 | .557 | 0.00 |
| CDR-SB | <0.01 | 1 | <0.01 | 0.15 | .701 | 0.00 |
| Age | 0.02 | 1 | 0.02 | 3.23 | .074 | 0.02 |
| Sex | <0.01 | 1 | <0.01 | 0.19 | .661 | 0.00 |
| Education | 0.01 | 1 | 0.01 | 1.47 | .226 | 0.01 |
| Scanner (GE dummy) | 0.01 | 1 | 0.01 | 1.84 | .177 | 0.01 |
| Scanner (Philips dummy) | 0.01 | 1 | 0.01 | 1.62 | .204 | 0.01 |
| Diagnosis | 0.02 | 1 | 0.02 | 2.85 | .093 | 0.02 |
| NPI-Q disinhibition | 0.01 | 1 | 0.01 | 0.60 | .439 | 0.00 |
| Diagnosis * NPI-Q disinhibition | 0.07 | 1 | 0.07 | 8.92 | .003 | 0.05 |
| Error | 1.30 | 172 | 0.01 |  |  |  |

*Note*. ICV=intracranial volume, CDR-SB=Clinical Dementia Rating Scale Sum of Boxes, NPI-Q= Neuropsychiatric Inventory Questionnaire, GE=General Electric

**Supplementary Table 2. Results of Salience Network Global efficiency 2 (diagnosis) x 2 (disinhibition presence) x 5 (threshold) repeated measures ANCOVA**

| Source | Type III Sum of Squares | | df | Mean Square | F | Sig. | Partial Eta Squared |
| --- | --- | --- | --- | --- | --- | --- | --- |
| Intercept | | 0.70 | 1 | 0.70 | 927.96 | <.001 | 0.84 |
| Estimated total ICV | | <0.01 | 1 | <0.01 | 3.76 | .054 | 0.02 |
| Days between MRI and NPI-Q | | <0.01 | 1 | <0.01 | 0.17 | .677 | <0.01 |
| CDR-SB | | <0.01 | 1 | <0.01 | 0.04 | .836 | <0.01 |
| Age | | <0.01 | 1 | <0.01 | 0.26 | .609 | <0.01 |
| Sex | | <0.01 | 1 | <0.01 | 0.18 | .670 | <0.01 |
| Education | | <0.01 | 1 | <0.01 | 4.97 | .027 | 0.03 |
| Scanner (GE dummy) | | <0.01 | 1 | <0.01 | 4.64 | .033 | 0.03 |
| Scanner (Philips dummy) | | <0.01 | 1 | <0.01 | 3.68 | .057 | 0.02 |
| Diagnosis | | <0.01 | 1 | <0.01 | 2.48 | .117 | 0.01 |
| NPI-Q disinhibition | | <0.01 | 1 | <0.01 | 4.70 | .032 | 0.03 |
| Diagnosis * NPI-Q disinhibition | | 0.01 | 1 | 0.01 | 8.06 | .005 | 0.05 |
| Error | | 0.13 | 172 | <0.01 |  |  |  |

*Note*. ICV=intracranial volume, CDR-SB=Clinical Dementia Rating Scale Sum of Boxes, NPI-Q= Neuropsychiatric Inventory Questionnaire, GE=General Electric

**Supplementary Table 3. Results of Cognitive Control Network Transitivity 2 (diagnosis) x 2 (disinhibition presence) x 5 (threshold) repeated measures ANCOVA**

| Source | Type III Sum of Squares | df | Mean Square | F | Sig. | Partial Eta Squared |
| --- | --- | --- | --- | --- | --- | --- |
| Intercept | 1.14 | 1 | 1.14 | 244.66 | <.001 | 0.59 |
| Estimated total ICV | <0.01 | 1 | <0.01 | <0.01 | .985 | <0.01 |
| Days between MRI and NPI-Q | <0.01 | 1 | <0.01 | 0.02 | .877 | <0.01 |
| CDR-SB | <0.01 | 1 | <0.01 | 0.09 | .763 | <0.01 |
| Age | <0.01 | 1 | <0.01 | 0.97 | .327 | 0.01 |
| Sex | <0.01 | 1 | <0.01 | 0.34 | .562 | <0.01 |
| Education | 0.01 | 1 | 0.01 | 1.66 | .199 | 0.01 |
| Scanner (GE dummy) | <0.01 | 1 | <0.01 | 0.66 | .418 | <0.01 |
| Scanner (Philips dummy) | 0.01 | 1 | 0.01 | 1.18 | .280 | 0.01 |
| Diagnosis | 0.02 | 1 | 0.02 | 4.30 | .040 | 0.02 |
| NPI-Q disinhibition | 0.05 | 1 | 0.05 | 10.53 | .001 | 0.06 |
| Diagnosis * NPI-Q disinhibition | 0.03 | 1 | 0.03 | 5.90 | .016 | 0.03 |
| Error | 0.80 | 172 | 0.01 |  |  |  |

*Note*. ICV=intracranial volume, CDR-SB=Clinical Dementia Rating Scale Sum of Boxes, NPI-Q= Neuropsychiatric Inventory Questionnaire, GE=General Electric

**Supplementary Table 4. Results of Cognitive Control Network Global Efficiency 2 (diagnosis) x 2 (disinhibition presence) x 5 (threshold) repeated measures ANCOVA**

| Source | Type III Sum of Squares | df | Mean Square | F | Sig. | Partial Eta Squared |
| --- | --- | --- | --- | --- | --- | --- |
| Intercept | 0.75 | 1 | 0.75 | 1684.29 | <.001 | 0.91 |
| Estimated total ICV | <0.01 | 1 | <0.01 | 0.23 | .635 | <0.01 |
| Days between MRI and NPI-Q | <0.01 | 1 | <0.01 | 0.17 | .678 | <0.01 |
| CDR-SB | <0.01 | 1 | <0.01 | 0.60 | .442 | <0.01 |
| Age | <0.01 | 1 | <0.01 | 0.87 | .351 | 0.01 |
| Sex | <0.01 | 1 | <0.01 | 0.09 | .764 | <0.01 |
| Education | <0.01 | 1 | <0.01 | 1.05 | .306 | 0.01 |
| Scanner (GE dummy) | <0.01 | 1 | <0.01 | 0.29 | .589 | <0.01 |
| Scanner (Philips dummy) | <0.01 | 1 | <0.01 | 0.51 | .475 | <0.01 |
| Diagnosis | <0.01 | 1 | <0.01 | 3.69 | .056 | 0.02 |
| NPI-Q disinhibition | <0.01 | 1 | <0.01 | 5.95 | .016 | 0.03 |
| Diagnosis * NPI-Q disinhibition | <0.01 | 1 | <0.01 | 3.16 | .077 | 0.02 |
| Error | 0.08 | 172 | <0.01 |  |  |  |

*Note*. ICV=intracranial volume, CDR-SB=Clinical Dementia Rating Scale Sum of Boxes, NPI-Q= Neuropsychiatric Inventory Questionnaire, GE=General Electric

**Supplementary Table 5. Results of Salience Network Transitivity 2 (diagnosis) x 2 (disinhibition presence) x 5 (threshold) repeated measures ANCOVA with race additionally covaried**

| Source | Type III Sum of Squares | df | Mean Square | F | Sig. | Partial Eta Squared |
| --- | --- | --- | --- | --- | --- | --- |
| Intercept | 0.91 | 1 | 0.91 | 122.33 | <.001 | 0.42 |
| Estimated total ICV | <0.01 | 1 | <0.01 | 0.26 | .610 | <0.01 |
| Days between MRI and NPI-Q | <0.01 | 1 | <0.01 | 0.33 | .569 | <0.01 |
| CDR-SB | <0.01 | 1 | <0.01 | 0.30 | .583 | <0.01 |
| Age | 0.03 | 1 | 0.03 | 3.50 | .063 | 0.02 |
| Sex | <0.01 | 1 | <0.01 | 0.04 | .849 | <0.01 |
| Education | 0.01 | 1 | 0.01 | 1.86 | .174 | 0.01 |
| Scanner (GE dummy) | 0.01 | 1 | 0.01 | 1.65 | .201 | 0.01 |
| Scanner (Philips dummy) | 0.02 | 1 | 0.02 | 2.08 | .151 | 0.01 |
| Black race (dummy) | <0.01 | 1 | <0.01 | 0.03 | .866 | <0.01 |
| Asian race (dummy) | 0.03 | 1 | 0.03 | 4.52 | .035 | 0.03 |
| Multiple races (dummy) | <0.01 | 1 | <0.01 | 0.38 | .538 | <0.01 |
| Diagnosis | 0.02 | 1 | 0.02 | 3.05 | .082 | 0.02 |
| NPI-Q disinhibition | <0.01 | 1 | <0.01 | 0.55 | .461 | <0.01 |
| Diagnosis * NPI-Q disinhibition | 0.06 | 1 | 0.06 | 8.62 | .004 | 0.05 |
| Error | 1.26 | 169 | 0.01 |  |  |  |

*Note*. ICV=intracranial volume, CDR-SB=Clinical Dementia Rating Scale Sum of Boxes, NPI-Q= Neuropsychiatric Inventory Questionnaire, GE=General Electric

**Supplementary Table 6. Results of Salience Network Global Efficiency 2 (diagnosis) x 2 (disinhibition presence) x 5 (threshold) repeated measures ANCOVA with race additionally covaried**

| Source | Type III Sum of Squares | df | Mean Square | F | Sig. | Partial Eta Squared |
| --- | --- | --- | --- | --- | --- | --- |
| Intercept | 0.69 | 1 | 0.69 | 905.96 | <.001 | 0.84 |
| Estimated total ICV | <0.01 | 1 | <0.01 | 3.55 | .061 | 0.02 |
| Days between MRI and NPI-Q | <0.01 | 1 | <0.01 | 0.21 | .649 | <0.01 |
| CDR-SB | <0.01 | 1 | <0.01 | <0.00 | .994 | <0.01 |
| Age | <0.01 | 1 | <0.01 | 0.28 | .598 | <0.01 |
| Sex | <0.01 | 1 | <0.01 | 0.17 | .682 | <0.01 |
| Education | <0.01 | 1 | <0.01 | 4.78 | .030 | 0.03 |
| Scanner (GE dummy) | <0.01 | 1 | <0.01 | 4.81 | .030 | 0.03 |
| Scanner (Philips dummy) | <0.01 | 1 | <0.01 | 3.96 | .048 | 0.02 |
| Black race (dummy) | <0.01 | 1 | <0.01 | 0.64 | .424 | <0.01 |
| Asian race (dummy) | <0.01 | 1 | <0.01 | 0.26 | .611 | <0.01 |
| Multiple races (dummy) | <0.01 | 1 | <0.01 | 0.24 | .625 | <0.01 |
| Diagnosis | <0.01 | 1 | <0.01 | 2.54 | .113 | 0.02 |
| NPI-Q disinhibition | <0.01 | 1 | <0.01 | 4.16 | .043 | 0.02 |
| Diagnosis * NPI-Q disinhibition | 0.01 | 1 | 0.01 | 7.75 | .006 | 0.04 |
| Error | 0.13 | 169 | 0.00 |  |  |  |

*Note*. ICV=intracranial volume, CDR-SB=Clinical Dementia Rating Scale Sum of Boxes, NPI-Q= Neuropsychiatric Inventory Questionnaire, GE=General Electric

**Supplementary Table 7. Results of Cognitive Control Network Transitivity 2 (diagnosis) x 2 (disinhibition presence) x 5 (threshold) repeated measures ANCOVA with race additionally covaried**

| Source | Type III Sum of Squares | df | Mean Square | F | Sig. | Partial Eta Squared |
| --- | --- | --- | --- | --- | --- | --- |
| Intercept | 1.12 | 1 | 1.12 | 239.65 | <.001 | 0.59 |
| Estimated total ICV | <0.01 | 1 | <0.01 | 0.01 | .947 | <0.00 |
| Days between MRI and NPI-Q | <0.01 | 1 | <0.01 | 0.01 | .925 | <0.00 |
| CDR-SB | <0.01 | 1 | <0.01 | 0.13 | .718 | <0.00 |
| Age | 0.01 | 1 | 0.01 | 0.99 | .322 | 0.01 |
| Sex | <0.01 | 1 | <0.01 | 0.23 | .631 | <0.01 |
| Education | 0.01 | 1 | 0.01 | 1.17 | .282 | 0.01 |
| Scanner (GE dummy) | <0.01 | 1 | <0.01 | 0.87 | .352 | 0.01 |
| Scanner (Philips dummy) | 0.01 | 1 | 0.01 | 1.20 | .276 | 0.01 |
| Black race (dummy) | <0.01 | 1 | <0.01 | 0.55 | .460 | <0.01 |
| Asian race (dummy) | <0.01 | 1 | <0.01 | 0.82 | .366 | 0.01 |
| Multiple races (dummy) | <0.01 | 1 | <0.01 | 0.03 | .862 | <0.01 |
| Diagnosis | 0.02 | 1 | 0.02 | 3.99 | .047 | 0.02 |
| NPI-Q disinhibition | 0.05 | 1 | 0.05 | 9.67 | .002 | 0.05 |
| Diagnosis * NPI-Q disinhibition | 0.03 | 1 | 0.03 | 6.12 | .014 | 0.04 |
| Error | 0.79 | 169 | 0.00 |  |  |  |

*Note*. ICV=intracranial volume, CDR-SB=Clinical Dementia Rating Scale Sum of Boxes, NPI-Q= Neuropsychiatric Inventory Questionnaire, GE=General Electric

**Supplementary Table 8. Results of Cognitive Control Network Global Efficiency 2 (diagnosis) x 2 (disinhibition presence) x 5 (threshold) repeated measures ANCOVA with race additionally covaried**

| Source | Type III Sum of Squares | df | Mean Square | F | Sig. | Partial Eta Squared |
| --- | --- | --- | --- | --- | --- | --- |
| Intercept | 0.74 | 1 | 0.74 | 1654.48 | <.001 | 0.91 |
| Estimated total ICV | <0.01 | 1 | <0.01 | 0.24 | .623 | <0.00 |
| Days between MRI and NPI-Q | <0.01 | 1 | <0.01 | 0.22 | .640 | <0.00 |
| CDR-SB | <0.01 | 1 | <0.01 | 0.63 | .429 | <0.00 |
| Age | <0.01 | 1 | <0.01 | 0.93 | .337 | 0.01 |
| Sex | <0.01 | 1 | <0.01 | 0.13 | .721 | <0.01 |
| Education | <0.01 | 1 | <0.01 | 1.49 | .225 | 0.01 |
| Scanner (GE dummy) | <0.01 | 1 | <0.01 | 0.44 | .508 | <0.01 |
| Scanner (Philips dummy) | <0.01 | 1 | <0.01 | 0.54 | .466 | <0.01 |
| Black race (dummy) | <0.01 | 1 | <0.01 | 0.47 | .494 | <0.01 |
| Asian race (dummy) | <0.01 | 1 | <0.01 | 0.77 | .382 | 0.01 |
| Multiple races (dummy) | <0.01 | 1 | <0.01 | 0.43 | .515 | <0.01 |
| Diagnosis | <0.01 | 1 | <0.01 | 3.33 | .070 | 0.02 |
| NPI-Q disinhibition | <0.01 | 1 | <0.01 | 5.08 | .025 | 0.03 |
| Diagnosis * NPI-Q disinhibition | <0.01 | 1 | <0.01 | 3.46 | .065 | 0.02 |
| Error | 0.08 | 169 | 0.00 |  |  |  |

*Note*. ICV=intracranial volume, CDR-SB=Clinical Dementia Rating Scale Sum of Boxes, NPI-Q= Neuropsychiatric Inventory Questionnaire, GE=General Electric
